# Challenges in Education for Visually Impaired Children in Ghana: A Path to Inclusion

**DOI:** 10.1155/ijpe/6653071

**Published:** 2025-05-22

**Authors:** Sylvester Kyei-Gyamfi, Frank Kyei-Arthur, Theophilus Kwabena Abutima, Ada Adoley Allotey, Henry Afrifa, Evans Sakyi-Boadu, Kwame S. Sakyi

**Affiliations:** ^1^Department of Children, Ministry of Gender, Children and Social Protection, Accra, Greater Accra Region, Ghana; ^2^Department of Environment and Public Health, University of Environment and Sustainable Development, Somanya, Eastern Region, Ghana; ^3^Department of Sociology and Social Work, University for Development Studies, Tamale, Northern Region, Ghana; ^4^Human Settlement Unit, Environmental Protection Authority, Accra, Greater Accra Region, Ghana; ^5^Centre for Migration Studies, University of Ghana, Legon, Greater Accra Region, Ghana; ^6^Department of Sustainable Development and Policy, University of Environment and Sustainable Development, Somanya, Eastern Region, Ghana; ^7^Research and Partnership Department, Center for Learning and Childhood Development, Accra, Greater Accra Region, Ghana

**Keywords:** children with visual impairments, Ghana, satisfaction, teachers, teaching and learning

## Abstract

This study examined children with visual impairments (CwVI) satisfaction with school facilities, CwVIs' reasons for their satisfaction or dissatisfaction with school facilities, and steps to enhance their inclusion in teaching and learning. Grounded in Bronfenbrenner's ecological systems theory (EST) (1979), the study employed a mixed-methods approach, including a survey of 288 CwVIs, 73 key informant interviews (KIIs), 14 focus group discussions (FGDs), observations, and documentary reviews to complement the findings. The results revealed that 60.8% of CwVIs were satisfied with their school facilities, while 39.1% were dissatisfied. Key factors contributing to satisfaction included the suitability of the school compound and classroom furniture arrangement, which minimized accidents. In contrast, unsafe compounds, overcrowded classrooms, and poorly arranged furniture disrupted navigation and participation, exposing vulnerabilities in their learning environment. The findings highlighted the vital role of family and community involvement in fostering inclusive attitudes and advocating for infrastructure and resource improvements. Families actively supporting these efforts can significantly enhance support systems for CwVIs. The study underscores the need for collaboration between families and schools to build cohesive support networks that address the academic and social needs of CwVIs, fostering an inclusive learning environment.

## 1. Introduction

Education is a fundamental right and a cornerstone for fostering inclusion and equality in society. However, children with visual impairments (CwVIs) in Ghana face significant challenges that hinder their access to quality education and equitable opportunities. These challenges are deeply rooted in their schooling experience, including inadequate learning materials, substandard infrastructure, insufficient government services, limited parental support, and teaching practices that fail to accommodate their unique needs. The consequences of these barriers extend beyond the classroom, affecting their academic progression, self-esteem, and overall social inclusion [1, 2].

This research stands out as one of the few studies focusing specifically on the lived experiences of CwVIs in Ghana, particularly within the context of selected schools nationwide. It originates from a broader initiative commissioned by Visio International and the Department of Children of the Ministry of Gender, which aimed to evaluate critical factors influencing the educational journeys of CwVIs. This initial research addressed essential themes, including the self-esteem of CwVIs, their access to teaching and learning materials, and the adequacy of government and organizational support [3]. It also delved into the perspectives of parents and guardians on the challenges they face in nurturing and supporting CwVIs.

Despite global commitments to inclusive education, Ghana's efforts to address the specific needs of CwVIs have not kept pace with interventions for their sighted peers. For instance, the Inclusive Education Policy of 2015 highlights a national commitment to inclusivity, but implementation has been hampered by resource constraints and limited teacher training [4, 5].

This disparity leaves many visually impaired children unable to participate fully in or contribute to society. This study seeks to provide actionable insights by examining the perspectives of CwVIs on their school facilities, their satisfaction levels, and their recommendations for improving inclusion. By amplifying the voices of these children, the research highlights their unique challenges, paving the way for evidence-based solutions to address the barriers impeding their education and social integration.

While previous studies have explored the broader experiences of children with disabilities, the specific needs and challenges faced by CwVIs remain underrepresented in academic discourse [2, 6, 7]. This gap in understanding hampers the development of targeted policies and practices that could improve educational outcomes and enhance the well-being of CwVIs in Ghana. Bronfenbrenner's ecological systems theory (EST) (1979) serves as the guiding framework to frame this inquiry. This theory provides a robust lens to analyse how interactions across various environmental systems influence the experiences and inclusion of CwVIs in Ghanaian schools [8]. Through this approach, the study addresses three key research questions: (a) Are CwVIs satisfied with their school facilities? (b) What are the reasons for CwVI satisfaction or dissatisfaction with their school facilities? (c) What measures should be implemented to enhance the inclusion of CwVIs in teaching and learning?

## 2. Theoretical Review: EST

Bronfenbrenner's EST [8] provides a robust framework for understanding the multifaceted and dynamic interactions that shape an individual's development and experiences. EST emphasizes that human behaviour and outcomes are influenced by a series of interconnected systems, including the microsystem, mesosystem, exosystem, macrosystem, and chronosystem. Each system represents a different level of influence, from immediate interpersonal interactions to broader societal and historical contexts.

In the context of education for CwVIs in Ghana, the microsystem includes the immediate environment of the child, such as their family, peers, teachers, and classroom settings. Challenges in this system might include insufficient parental involvement, a lack of braille or tactile learning materials, or teachers untrained in inclusive teaching practices [6, 7]. These factors directly affect CwVIs' learning experiences and their perceptions of inclusion.

The mesosystem encompasses the interactions between different microsystems, such as the relationship between home and school. For example, limited communication or collaboration between parents and teachers can hinder efforts to address the specific needs of CwVIs. A lack of coordinated support from both the family and school environment exacerbates the challenges faced by these children [6, 7].

The exosystem represents external environments that indirectly influence the child, such as government policies, the availability of resources for special education, and community attitudes toward disability. Inadequate government funding for inclusive education, poor infrastructure, and societal stigmatization of disabilities are significant barriers in this system. These external factors limit the capacity of schools to provide the necessary support for CwVIs.

At the broader level, the macrosystem includes cultural norms, societal values, and national priorities. In Ghana, cultural perceptions of disability often reinforce exclusion and limit opportunities for CwVIs. Additionally, the prioritization of resources for general education over special education reflects systemic inequities that undermine efforts toward inclusion.

Finally, the chronosystem captures the dimension of time, considering how changes in systems or events across a child's life impact their experiences. For instance, recent global emphasis on inclusive education, coupled with technological advancements, presents opportunities to improve the educational experiences of CwVIs. However, historical neglect and entrenched inequalities continue to pose significant challenges.

By applying the EST, this study illuminates the multilayered factors that influence the inclusion of CwVIs in Ghanaian schools. Understanding these dynamics is crucial for designing interventions that address not only the immediate barriers faced by CwVIs but also the broader systemic and cultural factors that perpetuate their exclusion. Through this lens, the study contributes to the discourse on inclusive education and offers pathways for ensuring that CwVIs are not left behind in Ghana's educational landscape.

## 3. Methods

### 3.1. Setting

The study was conducted in Ghana across selected districts within Visio International's program areas. These districts included Wa, Hohoe, Central Tongu, North Tongu, Akuapem North, Cape Coast, and Tano South, spanning five regions. Ghana, a lower-middle-income West African nation located along the Gulf of Guinea coast, has Accra as its capital. As of 2021, the country's population stood at 30.8 million, with a majority being female. Covering a land area of approximately 238,537 km^2^, Ghana is bordered by Burkina Faso to the north, the Gulf of Guinea to the south, Togo to the east, and Côte d'Ivoire to the west. While the country now comprises 16 administrative regions, the data collection for this study was conducted when it had 10 regions [9].

### 3.2. Design

This study is part of a broader mixed-methods research project undertaken by the Ministry of Gender, Children and Social Protection (MoGCSP) and Visio International. The overall project utilized a quota sampling technique to recruit 400 participants, with allocations proportionate to the number of CwVIs in selected schools. The study employed both qualitative and quantitative approaches. The qualitative component included key informant interviews (KIIs), focus group discussions (FGDs), observations, and document reviews. The quantitative aspect comprised a structured survey. This specific study focuses on survey questions that explore the satisfaction of CwVIs with their school facilities and the reasons for their responses. Additional qualitative insights were derived from responses to KII and FGD guides administered to CwVIs and their teachers. While the primary focus was on CwVIs, teachers were engaged to provide deeper context and explanations for the children's responses.

### 3.3. Population and Sample Size

The study population consisted of CwVIs aged 8–17 years, currently enrolled in schools within Visio International's program areas. Of the 400 sampled participants, 75 were selected from schools in Wa and Akuapem North, given their larger populations of CwVIs. The remaining five districts contributed 50 participants each. Eligibility criteria required that participants be aged 8–17 years, currently attending school, and identified as CwVIs. The sample size of 400 was determined using the formula:

- *n* = *z*2 · *p* · (1 − *p*)*c*2
- *n* = *c*2*z*2 · *p* · (1 − *p*)

where

- *z* = 1.96 (95% confidence level)
- *p* = 0.5 (probability of response)
- *c* = 0.05 (confidence interval)

The calculated sample size was 384, with an additional buffer of 16 to accommodate nonresponses, resulting in a total of 400 participants.

### 3.4. Study Instruments

#### 3.4.1. Survey

The survey was administered over 1 month, from September to October 2022, using structured questionnaires. Individual interviews were conducted within school premises, primarily in English, with translations into local languages (Ewe, Akuapem, or Fante) where necessary. The survey achieved a response rate of 72%. Key themes covered in the questionnaire included the educational needs of CwVIs, classroom safety, self-esteem, the availability of learning materials, service responsiveness, and assessments of daycare centers for CwVIs aged 0–3 regarding education, health, and life skills development.

#### 3.4.2. KIIs

A total of 73 KIIs were conducted across the seven districts. Respondents included caregivers, service providers (e.g., schools, eye clinics, and social services), partner agencies (PAs), and community-based organizations (CBOs) working with CwVIs. Additionally, 10 key implementing partners of Visio International were interviewed to evaluate program effectiveness, identify challenges, and capture lessons for future initiatives.

#### 3.4.3. FGDs

Fourteen (14) FGDs involving 150 CwVIs were conducted. The groups were segmented by gender to ensure balanced representation of children in each district, with each FGD comprising 8–12 participants. These discussions provided complementary qualitative insights, offering a deeper understanding of the lived experiences of CwVIs.

#### 3.4.4. Document Review and Stakeholder Consultation

A thorough review of relevant documents was undertaken, including national policies on CwVIs, surveys, census data, and reports from Visio International's strategic partners. Consultations with stakeholders, such as the MoGCSP, the Department of Children (DOC), district assemblies, and nongovernmental organizations (NGOs) working with CwVIs, supplemented the document review.

#### 3.4.5. Observations

Direct observations were conducted to evaluate school facilities, social services, and household conditions in the communities of CwVIs. These observations provided firsthand insights into the educational and living environments of participants.

### 3.5. Measurement

#### 3.5.1. Dependent Variable

The main dependent variable for this study was satisfaction with school facilities. This was measured by asking CwVIs a single question: “Are you satisfied with your school facilities (e.g. classroom environments, school compound, availability of teaching and learning facilities, etc.)?” The responses were yes and no. A “yes” response means CwVIs are satisfied with school facilities, while a “no” response means dissatisfaction with school facilities.

#### 3.5.2. Independent Variables

The independent variables used in this study were the sociodemographic of CwVIs. Sex of CwVIs was categorized as “male” and “female” and age as “8–10 years,” “11–13 years,” and “14–17 years.” Also, the educational level of CwVIs was recategorized into “primary,” “junior high school (JHS),” and “senior high school (SHS)/vocational and higher education.” Furthermore, the religion of respondents was categorized as “Christianity,” “Islam,” “African traditional religion,” and “no religion.” Additionally, CwVIs in seven schools were interviewed: “Akuapem school,” “Wa school,” “Cape Coast school,” “Hohoe school,” “South Tongu school,” “North Tongu school,” and “Tano South school.”

### 3.6. Statistical Analysis

#### 3.6.1. Quantitative Data Analysis

Quantitative data were analysed using descriptive statistics (frequencies and percentages) and bivariate analyses through cross-tabulations to examine relationships between variables. Specifically, frequencies and percentages described the sociodemographic characteristics of CwVIs. Pearson's chi-square test and Fisher's exact test assessed associations between CwVIs' satisfaction with school facilities and their sociodemographic characteristics. Statistical analyses were performed using SPSS Version 26, with statistical significance determined at the 95% confidence level.

#### 3.6.2. Qualitative Data Analysis

**Figure 1:**
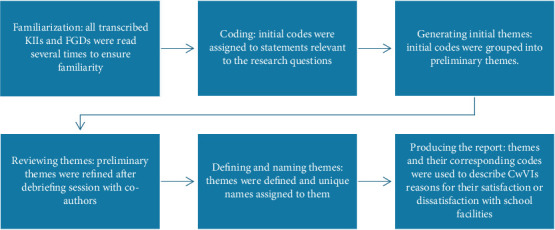
Steps for analysing the qualitative data.

Qualitative data from KIIs and FGDs were audio-recorded, transcribed verbatim, and analysed using thematic analysis. The analysis employed QSR NVivo Software Version 10 to identify key patterns and themes. Thematic analysis followed Braun and Clarke's reflexive thematic analysis (RTA) approach (2006), which allows for a flexible and iterative exploration of qualitative data while acknowledging the researchers' subjective perspectives [10]. The analysis of the qualitative data followed six key steps (refer to Figure 1). In the first stage of familiarization, the first author read all transcribed KIIs and FGDs several times to ensure familiarity with the data. The next step involved the systematic coding of statements relevant to the research questions. The first author identified statements in each transcript that were relevant to the research questions and assigned initial codes to these statements. Subsequently, the first author organized the initial codes into preliminary themes. Furthermore, to ensure rigor of the qualitative findings, a debriefing session was held with the remaining authors who have expertise in qualitative data analysis, and the preliminary themes were refined based on their feedback. During the debriefing session, similar themes were combined. Furthermore, during the debriefing session, themes were defined, and unique names were assigned to them. Moreover, the themes and their corresponding codes were used to describe experiences of CwVIs and teachers regarding the reasons for satisfaction and dissatisfaction with school facilities and steps needed to enhance inclusive learning environments for CwVIs. Another measure implemented to ensure the rigor of the qualitative findings was the documentation of all decisions taken during the data collection and the various stages of the data analysis.

The RTA approach ensured a thorough and reflective analysis of the qualitative data, supported using QSR NVivo Software Version 10 for thematic coding. This mixed-methods analysis provided a comprehensive understanding of the challenges and opportunities faced by CwVIs, as well as the reasons for their satisfaction or dissatisfaction with the facilities in their schools.

## 4. Results

### 4.1. Sociodemographic Characteristics of CwVIs

Table 1 displays the sociodemographic features of CwVIs, including sex, age, educational background, religion, and schools attended by respondents. The total number of CwVIs sampled was 288, with males comprising the majority (58.3%) and females constituting the minority (41.7%). The majority of the children (66.0%) were between the ages of 14 and 17, followed by those aged 11–13 (20.1%) and 8–10 (13.9%). The mean age was 8.25 years. In terms of education, a sizable number of CwVIs (60.1%) were in primary school, with 33.7% in JHS and 6.3% in SHS or higher education. The majority of CwVIs identified as Christians (85.4%), with Muslims accounting for 13.5%. A small minority (0.3%) practiced African traditional religion, while 0.7% claimed no religion. The CwVIs sampled were spread across several schools, with the Akuapem school having the most participants (21.2%), followed by Cape Coast (17.4%), Wa (14.2%), Tano South (13.9%), Hohoe (11.1%), North Tongu (11.5%), and South Tongu (10.8%).

### 4.2. Satisfaction With School Facilities

The study assessed CwVI satisfaction with school facilities across sex, age, and educational level.  Table 2 indicates that 60.8% of respondents rated satisfaction with their educational facilities, while 39.1% conveyed dissatisfaction. There was no statistically significant difference in satisfaction between males (60.7%) and females (60.8%) (*p* = 0.984). A significant difference was observed (*p* = 0.006), with younger children (8–10 years: 75.0% and 11–13 years: 72.4%) reporting higher satisfaction than older children (14–17 years: 54.2%). Satisfaction varied significantly by education level (*p* ≤ 0.001). Students in primary school (67.6%) and SHS (77.8%) were more satisfied with their school facilities compared to JHS students (45.4%).

### 4.3. Reasons for Satisfaction With School Facilities

Data in Table 3 reflects perspectives from both male and female CwVIs. For the children who expressed satisfaction, 30.9% found the school compound comfortable and suitable for their movements; 26.7% appreciated the proper arrangement of classroom furniture, which reduced accidents; and 3.8% expressed satisfaction due to having an adequate number of teachers for instruction. A range of factors contributed to the children's dissatisfaction, including inadequate teaching and learning facilities (17.7%), poor school furniture and infrastructure (10.8%), overcrowding in classrooms (4.9%), broken classroom furniture (2.8%), and an unfavourable school environment (2.4%).

The FGDs and KIIs provided diverse viewpoints and comprehensive explanations of the reasons children expressed dissatisfaction with their school facilities during the survey. These are grouped into (a) unsafe school compounds, (b) overcrowding classrooms, (c) poorly arranged classroom furniture, (d) inadequate teaching and learning materials, and (e) inadequate resource teachers for the CwVIs.

#### 4.3.1. Unsafe School Compounds

Both CwVIs and teachers made some remarks on why they felt CwVIs were not satisfied with their school facilities, with some reporting unsafe compounds. Some teachers reported the following:

Their surroundings are not ideal since erosion has formed little gutters in which they occasionally fall. During the rainy season, they find it more difficult to move around due to the slippery surfaces. The slippery surfaces increase the potential of accidents, so they must exercise caution. Furthermore, a lack of effective drainage exacerbates flooding in many areas, affecting the children's routines. (KII 1, teacher)

The campus environs are dangerous for them; the topography is unsuitable, and open gutters present a challenge. Occasionally, in their rush, they may clash. In truth, the school's initial design did not account for visual impairments, resulting in an infrastructure that is usually unfavourable to those with disabilities. This error demonstrates the critical need for upgrades that prioritise accessibility and safety for all students. (KII 2, teacher)

Some CwVIs added

Many times, students have bumped into each other, tripped over an object, or fallen by stepping into a gutter. I have been a victim several times. Each incident often leads to laughter among friends, but it can also be quite embarrassing. These moments serve as a reminder to always pay attention to our surroundings, even in the most familiar places. (FGD 1, boy 13 years)

We face many problems on campus. Open gutters, gullies, bushy environments, and poorly structured buildings surround us, posing varying dangers for children with visual problems like ours. These issues not only hinder our daily activities but also create an unsafe atmosphere that can affect our overall well-being. It is crucial for the administration to address these concerns and implement solutions that ensure a safer and more accessible environment for all students. (FGD 2, girl 15 years)

#### 4.3.2. Overcrowding Classrooms

The CwVIs indicated that there are more students than expected, creating overcrowding in their classrooms. One CwVI reported the following:

The overcrowding in the classrooms, which forces us to share cramped spaces and disrupts our concentration during lessons, makes sitting uncomfortable. This situation not only hampers our ability to focus but also limits opportunities for meaningful interaction with our peers. Many students experience disengagement and frustration as a result, which ultimately affects their overall academic performance. (FGD 3, boy 14 years)

A teacher reported that teaching and learning are always a challenge when the children are squeezed together in class:

Classes fall into confusion when the children sometimes must compete for space in class, which is not good for their learning. Class sometimes is interrupted when we must ensure the children sit comfortably and quietly. (KII 3, teacher)

#### 4.3.3. Poorly Arranged Classroom Furniture

Confirming the survey results, the issue of poor arrangements of class infrastructure was again mentioned during the FGDs with the CwVIs.

We should regularly arrange the desks and chairs in our classrooms before class each day, but this hasn't happened. Often, the placement of furniture in the walkway leads to children bumping into it during class. This not only disrupts the learning environment but also poses safety hazards for the students. To address this, it is essential to establish a classroom setup routine and involve students in maintaining a tidy and organized space. (FGD 4, boy 17 years)

#### 4.3.4. Inadequate Teaching and Learning Materials

The FGDs with the CwVIs revealed that many schools face challenges such as the unavailability, inadequacy, poor condition, outdated nature, excessive cost, and irregular supply of teaching and learning materials.

Attending school and feeling comfortable with learning pose a significant challenge for CwVIs. This is due to the constant scarcity of learning materials. Regularly, we experience shortages of brail sheets. Most of our Perkins braille and recorders have broken down, and we have no idea when they will be fixed or replaced with new ones. (FGD 5, boy 16 years)

Sir, sometimes I feel so sad because I keep wondering why we are always in short supply of learning materials when sighted schools are not facing the same situation. Currently, the insufficiency of these materials forces us to rely on hand frames and styluses, thereby decelerating our learning process. In almost all classes, there are either very few or no pencils available for students to use. (FGD 6, boy 17 years)

During the KIIs, some teachers had the following to say about the inadequate teaching and learning materials:

We have a limited number of braille materials, recorders, and braille sheets. Students often must pair up or wait their turn to use the few available resources, which significantly affects teaching and learning. What makes it worse is the impact on the children—many become demotivated due to these dire conditions. (KII 4, teacher)

Considering the diversity among the children, we require a substantial number of resources to help them catch up with their sighted peers. These include voice recorders, magnifiers for students with low vision, and Braillers. Previously, we had about 20 Perkins Braillers provided by the government, but they have all broken down, and we are unable to repair them due to the unavailability of spare parts. The Perkins Brailler is a fundamental tool for these students. In its absence, they are forced to rely on hand frames and styluses, which slow down their writing pace significantly. Additionally, we need tactile materials to enable children to use their sense of touch for identification. Without these, we are left to rely on descriptions, which often include abstract concepts that do not effectively aid their learning. (KII 5, teacher)

#### 4.3.5. Inadequate Resource Teachers for the CwVIs

Teachers highlighted the insufficient availability of resource teachers in schools during the KIIs. Some of the concerns raised are as follows:

The resource teachers are not enough. Ideally, each class should have a resource teacher. The shortage of resource teachers significantly impacts the support that students with disabilities receive, hindering their educational progress. Special education requires that every student receives the attention they need to thrive academically. (KII 6, teacher)

The GES policy requires a teacher to handle eight students. However, the number of students we currently manage outnumbers the number of teachers, making it difficult for us to manage the workload, which presents its own set of issues. Currently, several teachers here have no prior experience in special education, so they are learning on the job, which is tough because braille is a technical subject that requires specialize training. If a teacher does not receive that training, it is exceedingly difficult to learn on the job. (KII 7, teacher)

Regarding reasons for satisfaction with school facilities, five themes merged during the FGDs and KIIs with CwVIs and teachers. The reasons include (a) availability of instructional staff in schools, (b) safe classroom furniture arrangement to prevent accidents, and (c) mobility-friendly school compounds for CwVIs. However, we also noted two additional reasons: (d) support for developing musical talents in schools and (e) availability of leisure, recreation, and entertainment facilities.

#### 4.3.6. Availability of Instructional Staff in Schools

Some CwVIs expressed their satisfaction with their schools due to the presence of adequate teachers:

Although there is room for improvement, the current number of teachers in our school is sufficient. Most of the work falls to students, not teachers. The teacher's role is to guide us, and with more effort, students can use the teacher's instructions to pass the exams. (FGD 7, girl 15 years)

#### 4.3.7. Safe Classroom Furniture Arrangement to Prevent Accidents

Some schools had better classroom furniture arrangements than others, suggesting that not all schools had inadequate classroom arrangements. The CwVIs shared the following observations:

An NGO donated a modern set of furniture to this school. Our school boasts classroom spaces large enough to accommodate all furniture, arranged in an orderly manner that promotes children's safe movement in class. This thoughtful arrangement not only enhances our overall learning environment but also significantly reduces the risk of accidents in school. It also makes us children feel more comfortable and focused on our schoolwork. (FGD 8, girl 17 years)

#### 4.3.8. Mobility-Friendly School Compounds for CwVIs

Some schools have made provision for the safe movements of CwVIs on their school's premises. They have a safe and clear passageway where no objects are in the way for CwVIs to bump into. One CwVI shared this sentiment:

Our school covers all gutters to prevent students from falling into them. The administration prioritises the safety of children on campus. By implementing such measures, schools demonstrate their commitment to accessibility and support for all students, promoting a sense of safety while in school. (FGD 9, girl 13 years)

#### 4.3.9. Support for Developing Musical Talents in Schools

Some CwVIs were happy with her school because the school facilities helped them develop their talents. Two CwVIs made the following observations:

I am a talented guitarist and can compose songs. The presence of our school band, band leader, and colleagues has greatly improved my musical talents. Many children like me have also benefitted from the school band. (FGD 10, boy 17 years)

I'm learning to weave and do various crafts, and our art tutor is quite helpful. He is patient and guides us, which serves as a powerful motivator. His encouragement fosters a pleasant classroom environment, allowing us to express our ideas without fear of making mistakes. With each session, I feel more secure in my abilities and eager to take on new assignments. (FGD 11, girl 16 years)

#### 4.3.10. Availability of Leisure, Recreation, and Entertainment Facilities

CwVIs explained that the presence of facilities for leisure, recreation, and entertainment makes them satisfied and content in school, compared to home, where they may not have the same facilities and friends to entertain with.

The availability of leisure, recreational, and entertainment facilities in school makes school a joyful experience. Indeed, once I arrive at school, I don't feel the need to return home. I have all the facilities to entertain and friends to play with. (FGD 12, boy 11 years)

### 4.4. Measures to Enhance Inclusion of CwVIs in Teaching and Learning

The study aimed to engage CwVIs and teachers to gain insights into their viewpoints on measures to improve CwVI inclusion in school during the FGDs. They deemed several recommendations crucial for fostering equal opportunities and social integration. These include (a) improving physical accessibility, (b) providing adaptive technology, (c) supplying educational materials, (d) providing in-service training for teachers and support staff, and (e) enhancing parental and community engagement.

#### 4.4.1. Physical Accessibility

CwVIs requested schools to prioritize physical accessibility by ensuring that school compounds are designed for the safety of CwVIs. This includes covering of gutters which poses a danger for CwVIs falling and having wide and free corridors to facilitate easy navigation of CwVIs' movements. The following were recommended:

The gutters on the school compound that occasionally makes children fall should all be covered, as well as all structures that are in the way of children and making movements on campus inhibitive should be properly calibrated. (FGD 13, boy 14 years)

There should be free-flow walking passages having wide and free corridors to facilitate easy navigation of children's movements. This will prevent child colliding with one another or bumping into objects. (FGD 14, girl 15 years)

Some recommendations were also made regarding classroom infrastructural arrangements:

We spent a great deal of time in the classroom areas, regularly darting out for the washroom, break or dining hall. The furniture should be arranged in such a way as to facilitate our free movement due to our visual challenges. (FGD 15, girl 17 years)

I believe it is about time the government build us state-of-the-art classroom infrastructure like that being done in sighted schools. Such infrastructure will surely solve the challenges we face with accessibility in school. (FGD 16, girl 17 years)

#### 4.4.2. Provision of Adaptive Technology

The FGDs with CwVIs also revealed some steps that ought to be undertaken to enhance inclusion in teaching and learning in the schools. One CwVI summed up indicating

The government should invest as early as possible in adaptive technology to support visual impairment. In a modern era like this, when we are still using arcade facilities is not helping us. We are lagging while the rest of the world is moving fast ahead in learning. We need assistive software, such as screen readers and magnification tools. We need modern computer labs with multimedia materials and equipped with accessible digital platforms for online learning. (FGD 17, boy 15 years)

#### 4.4.3. Supply of Educational Materials

CwVIs suggested the need for schools to make regular provision of accessible educational materials. Below are quotes from CwVIs to buttress the point:

Most of the materials we use are specialised materials. Unfortunately, as our numbers keep increasing due to enrolment as the years go by, the increasing population is not commensurate the materials we need for learning. Government and its partners, including civil society should come to our aid by supplying ⁠Braille, large print texts, tactile materials, audio books, recorders, talking calculators, and accessible digital materials. This is the only to improve inclusive education to catch up with our sighted colleagues. (FGD 17, girl 16 years)

Despite our vision impairment and need for more learning aids in school, sighted children have more instructional materials than us. Our teaching and learning suffer because we consistently fall behind in the educational demands, thereby lowering our educational standards. Inclusion requires equal opportunities for both CwVIs and sighted children. To truly foster an inclusive environment, schools must prioritize equitable access to resources and support for all students, regardless of their visual abilities. (FGD 18, boy 16 years)

Some teachers added:

Visually challenged children struggle with basic education, and their requirements are frequently unfulfilled. Many people believe they are second-class citizens, which limits their integration into society and decreases their self-esteem. The most successful strategy is to provide a steady supply of teaching and learning resources. (KII 6 with a teacher)

#### 4.4.4. Provision of In-Service Training for Teachers and Support Staff

Teachers suggested regular capacity building for teachers through in-service training and other course to upgrade their knowledge and skills to handle visual impairment concerns.

Schools that enrol CwVIs should ensure that their personnel have acquired the required training to assist children with visual impairments. This training should cover effective communication techniques, classroom accommodations, and the use of assistive technology. Furthermore, creating an inclusive environment would improve the educational experience for all students by encouraging empathy and understanding among classmates. (KII 8, teacher)

#### 4.4.5. Enhance Parental and Community Engagement

Support from parents, families, and the community has significant implications for the inclusive education of CwVIs. The KIIs and FGDs with teachers and CwVIs revealed that many of the CwVIs would be pleased if the attitudes and treatments given to them in their homes and communities were improved to be more inclusive. The following were expressed:

Family members should treat us as individuals with disabilities but also as normal human beings, just like those without disabilities. This will enhance our self-esteem and boost our interest in school, which will benefit our academic performance. (FGD 19, girl 16 years)

For their benefit, the acceptance of CwVIs at home and in communities should improve. This acceptance can foster an environment where children can thrive and develop their unique talents. By creating spaces that celebrate diversity and promote understanding, we can empower these children to reach their full potential, both academically and socially. (KII 9, teacher)

Schools should foster partnerships with organisations in the various communities supporting visual impairment and engage with the local community. This includes support for infrastructural development, supply of teaching and learning materials, and sponsorships for in-service training teachers. (KII 10, teacher)

## 5. Discussion

This study examined CwVI satisfaction with their school facilities, their reasons for the satisfaction or dissatisfaction with school facilities, and the steps required to enhance their inclusion in teaching and learning. The findings provide critical insights into the experiences of CwVIs in Ghanaian schools, particularly regarding their interactions with school facilities and the challenges they encounter. Bronfenbrenner's EST serves as an effective framework for analysing these findings, illustrating how individual development is shaped by environmental and systemic factors. EST's five interconnected levels—the microsystem, mesosystem, exosystem, macrosystem, and chronosystem—underscore the various layers of influence affecting CwVIs in educational settings [8, 11].

At the microsystem level, findings revealed mixed satisfaction levels among CwVIs regarding school facilities, with 60.8% expressing satisfaction and 39.1% reporting dissatisfaction. This level focuses on daily interactions between CwVIs and their immediate environments, including peers, teachers, and the physical school infrastructure. Factors such as the suitability of the school compound (30.9%) and the arrangement of classroom furniture to minimize accidents (26.7%) were key contributors to satisfaction. These findings align with literature emphasizing the importance of safe and supportive environments for children with disabilities [12]. Conversely, unsafe school compounds, overcrowded classrooms, and poorly arranged furniture disrupted students' navigation and participation, highlighting vulnerabilities in their immediate environment [13].

The mesosystem level reflects the interactions between multiple microsystems, such as the home and school environments. Our findings underscore the critical role of family and community involvement in fostering inclusive attitudes and advocating for necessary improvements. For instance, families actively supporting infrastructure enhancements or resource provision significantly strengthened the support systems for CwVIs. This highlights the need for collaborative efforts between families and schools to establish a cohesive support network, addressing the diverse academic and social needs of CwVIs [13]. Enhancing communication and partnerships between these microsystems can create more inclusive educational experiences.

The exosystem encompasses external factors that indirectly affect CwVIs' experiences, including policies, resource allocation, and teacher training. The study revealed systemic issues such as a lack of Braille sheets, broken Perkins braillers, and outdated materials, all of which stem from inequities in resource distribution. These challenges underline the need for equitable resource allocation to support inclusive education [14]. Additionally, the inadequate number of specialized teachers further exacerbates barriers to inclusion, aligning with global findings that insufficient teacher training is a significant hindrance [6, 15].

At the macrosystem level, societal attitudes and cultural norms heavily influence the educational experiences of CwVIs. The study highlighted the absence of inclusive infrastructure designs, reflecting a broader societal neglect of accessibility. Positive developments, such as the integration of mobility-friendly features like covered gutters and widened walkways, signal a growing acknowledgment of accessibility as a critical component of education [16]. However, the demand for assistive technologies and consistent provision of learning aids may suggest the need for sustained advocacy and policy reform by institutions such as the MoGCSP. These measures not only improve educational outcomes for CwVIs but also promote equity and inclusion within the broader educational system.

Finally, the chronosystem emphasizes the influence of time, capturing changes in policies, resources, and societal attitudes over time. Participants' calls for continuous investment in adaptive technologies and in-service teacher training reflect the dynamic nature of inclusive education. Addressing these systemic gaps through sustained efforts can lead to long-term improvements in the learning experiences of CwVIs, fostering a culture that prioritizes equity and inclusion [17].

## 6. Limitations and Strengths

This study demonstrates considerable strengths, particularly its implications for enhancing teaching and learning practices within the special education sector. It provides valuable insights into the experiences of CwVIs in school, contributing to a better understanding of their educational needs. However, the study's small sample size limits the generalizability of the findings to broader populations. Additionally, the cross-sectional design precludes the ability to establish causal relationships between variables. Another limitation is that the study predominantly focused on CwVIs and teachers in mixed schools, where children with and without visual impairments coexist. The exclusion of opinions from children without disabilities may introduce bias, as their perspectives could have served as a cross-check to validate or challenge the views expressed by CwVIs. Moreover, using a single question to measure CwVIs' satisfaction with school facilities and its binary response does not adequately capture the various elements that constitute school facilities and the dimensions of satisfaction associated with these facilities for a nuanced analysis. Future studies should assess each element of school facilities and utilize a Likert scale to measure the diverse dimensions of satisfaction with each element.

## 7. Conclusion

This study sheds light on the dual realities faced by CwVIs in Ghanaian schools. While many expressed appreciations for certain facilities and support mechanisms, systemic challenges such as unsafe environments, overcrowding, and inadequate teaching resources continue to hinder their learning experiences. Using Bronfenbrenner's EST as a guiding framework reveals the need for a multifaceted approach to address these challenges across all levels of influence. To enhance inclusivity and improve educational outcomes for CwVIs, the study recommends prioritizing infrastructure upgrades to ensure physical accessibility, investing in adaptive technologies, and maintaining a consistent supply of specialized educational materials. Regular in-service training for teachers, alongside active community and parental engagement, is essential for creating a supportive environment that empowers CwVIs to thrive academically and socially. By addressing these challenges, stakeholders can improve not only the quality of education for CwVIs but also promote broader equity, inclusion, and dignity within the school system. These actions have the potential to transform Ghana's educational landscape, ensuring that every child, regardless of ability, has access to quality education in an inclusive environment.

## Figures and Tables

**Table 1:** Sociodemographic characteristics of CwVIs.

	**Frequency**	**Percent**
Sex		
Male	168	58.3
Female	120	41.7
Age		
8–10 years	40	13.9
11–13 years	58	20.1
14–17 years	190	66.0
Mean age		8.25
Educational level		
Primary	173	60.1
JHS	97	33.7
SHS/vocational and higher	18	6.3
Religion		
Christianity	246	85.4
Islam	39	13.5
African traditional religion	1	0.3
No religion	2	0.7
School		
Akuapem school	61	21.2
Wa school	41	14.2
Cape Coast school	50	17.4
Hohoe school	32	11.1
South Tongu school	31	10.8
North Tongu school	33	11.5
Tano South school	40	13.9
Total	288	100

**Table 2:** Satisfaction with school facilities by sex, age, and educational level.

**Response**	**Yes (%)**	**No (%)**	**p** ** values**
Sex			0.984
Male	102 (60.7)	66 (39.3)	
Female	73 (60.8)	47 (39.2)	
Age			0.006
8–10 years	30 (75.0)	10 (25.0)	
11–13 years	42 (72.4)	16 (27.6)	
14–17 years	103 (54.2)	87 (45.8)	
Educational level			< 0.001
Primary	117 (67.6)	56 (32.4)	
JHS	44 (45.4)	53 (54.6)	
SHS	14 (77.8)	4 (22.2)	
Total	175 (60.8)	113 (39.1)	

**Table 3:** Reasons for CwVIs being satisfied or unsatisfied with school facilities.

**Reason**	**Male (%)**	**Female (%)**	**Total (%)**	**p** ** value**
Availability of teachers for instructions	4 (2.4)	7 (5.8)	11 (3.8)	0.606
Classroom furniture arrangement done to prevent	47 (28.0)	30 (25.0)	77 (26.7)	
Classrooms have broken furniture	4 (2.4)	4 (3.3)	8 (92.8)	
School compound is very comfortable and suitable for the movements of CwVIs	53 (31.5)	36 (30.0)	89 (30.9)	
State of school furniture and infrastructure is very bad and unsuitable for CwVIs	16 (9.5)	15 (12.5)	31 (10.8)	
School environments are not suitable for CwVIs' movements	6 (3.6)	1 (0.8)	7 (2.4)	
School has inadequate teaching and learning facilities	30 (17.9)	21 (17.5)	51 (17.7)	
Classrooms are overcrowded	8 (4.8)	6 (5.0)	14 (4.9)	

## Data Availability

The data that support the findings of this study are available on request from the corresponding author. The data are not publicly available due to privacy or ethical restrictions.
